# Pain assessment and management of adult patients in the Swedish EMS: a nationwide registry study

**DOI:** 10.1186/s13049-025-01333-2

**Published:** 2025-02-05

**Authors:** Glenn Larsson, Pär Wennberg, Kristoffer Wibring

**Affiliations:** 1 PICTA, Prehospital Innovation Arena, Lindholmen Science Park, Gothenburg, Sweden; 2 PreHospen – Centre for Prehospital Research, University of Borås, Borås, Sweden; 3 Research, Education, Development and Innovation Department, Skaraborg Hospital, Skövde, Sweden; 4 School of Health Sciences, Jönköping University, Jönköping, Sweden; 5 School of Health Sciences, University of Skövde, Skövde, Sweden; 6 Department of Ambulance and Prehospital Care, Halmstad, Region Halland Sweden; 7 Institute of Health and Care Sciences, Sahlgrenska Academy, University of Gothenburg, Gothenburg, Sweden

**Keywords:** Pain, Pain assessment, Pain management, Prehospital, Emergency medical services, Ambulance services

## Abstract

**Background:**

Pain is a frequent reason for contacting the Emergency Medical Services (EMS), and effective pain management constitutes one of its cornerstones. The aims of this study have been: (a) to describe the prevalence of pain intensity ratings in EMS care of patients with pain-related conditions; (b) to describe pain treatment in the EMS setting in terms of drugs administered and the proportion of patients receiving analgesics and (c) to investigate the relationship between patients’ self-reported pain intensity and vital signs.

**Methods:**

This is a retrospective observational cohort study using data from 394,700 EMS missions conducted 2021 and 2022, as recorded in the Swedish Ambulance Registry. The study focused on patients who contacted the EMS due to pain, trauma, or injury. Pain intensity was recorded using the Numeric Rating Scale (NRS). NRS scores of 5–10 were considered as high-level pain and NRS ≤ 4 as low-level. Descriptive statistics were used to present categorical and continuous variables. Chi-square tests were applied for dichotomous variables, while Kruskal–Wallis tests were used for ordinal data. Logistic regression analysis was carried out to identify factors associated with pain intensity and analgesic treatment. *p* value < 0.001 was considered statistically significant.

**Results:**

Pain intensity was recorded in 23.6% of cases. Most patients rated their pain as high-level (NRS 5–10, 57.4% of those assessed). Analgesics were administered in 27.5% of cases, with higher administration rates observed when pain intensity was documented. Female sex, higher breathing rates, and higher systolic blood pressure were associated with higher pain intensity, while increasing age was associated with lower odds of reporting high-level pain intensity. No significant association was found between heart rate and pain intensity.

**Conclusion:**

This 2-year cohort study highlights significant deficiencies in recorded pain assessment and management in the Swedish EMS. Only 22.5% of the patients had their pain assessed with a validated scale, while 27.5% received analgesics, although pain-related conditions were a common reason for contacting the EMS. The findings indicate a lack of systematic pain assessment which puts many patients at risk of insufficient pain relief.

**Supplementary Information:**

The online version contains supplementary material available at 10.1186/s13049-025-01333-2.

## Background

Pain is a common cause of contact with the Emergency Medical Services (EMS) [1, 2] and its management is one of the cornerstones of EMS care [3]. Pain management encompasses pain assessment, pain treatment, and evaluation of the effectiveness of the treatment [4]. The unpredictable nature of the EMS context, such as limited information regarding the cause of the medical condition or injury, insufficient knowledge of the patient’s medical history, and a dynamic and often challenging environment can complicate pain management [5]. Validated instruments for assessing pain intensity, such as the Numeric Rating Scale (NRS) and the Visual Analogue Scale (VAS), should be used [6]. Other rating systems, such as the Behaviour Rating Scale (BRS), have been applied in previous EMS research, but these scales lack sufficient validation [7]. Incomplete or missing documentation of pain intensity suggests that pain assessment remains a challenge in the EMS context [8].

The effectiveness of prehospital pain management depends on several factors, such as the duration of the EMS mission [7, 9], the presence of pain assessment [7], the type of pain [10], the intensity of the initial pain [11], and the priority with which the mission is dispatched [8]. Furthermore, some studies indicate weak correlations between pain intensity and physiological parameters such as respiratory rate [8, 12]. Inadequate pain assessment and treatment can lead to complications, including increased anxiety, prolonged hospital stays, and reduced quality of life [4].

Patients cared for by the EMS do not receive pain relief to the extent that would be desirable [10, 13]. Various conditions result in different levels of pain management, and although pain levels may vary according to the condition, this does not appear to explain the variation in pain relief [9]. Even among patients reporting severe pain, the level and effectiveness of treatment show significant inconsistencies [7].

There is insufficient knowledge regarding the relationship between documented pain intensity and treatment outcomes, as well as variation in pain management practices across different patient conditions [4, 9]. These knowledge gaps, combined with conflicting findings from previous studies, highlight the need for further research to enhance our understanding of pain management in the EMS. The Swedish Ambulance Registry (AmbuReg) provides an opportunity to carry out a nationwide review of pain management practices in Swedish EMS care.

## Methods

### Aim

- to describe the prevalence of pain intensity ratings in EMS care for patients with pain-related conditions,
- to describe pain treatment in the EMS setting in terms of drugs administered and the proportion of patients receiving analgesics, and
- to investigate the relationship between the patients’ self-rated pain intensity and vital signs.

### Study design

This is a retrospective observational cohort study based on national quality registry data from AmbuReg.

### Setting and population

The population of Sweden is approximately 10.3 million, and the country covers an area of 450,295 km^2^, divided into 21 regions. The EMS are tax-funded, and each region organises its EMS independently. Approximately 1 million primary EMS missions are conducted annually, with around 75% of patients are transported to emergency departments and 25% are non-conveyed [14].

According to Swedish legislation, all ambulances must be staffed by at least one registered nurse (RN) authorized to assess patients’ conditions and administer pharmacological treatments. Many EMS organisations additionally require RNs to complete a 1-year master’s program in prehospital emergency care. Typical EMS crew configurations consist of either two nurses or one nurse paired with an emergency medical technician (EMT) [15]. There are several options for pharmacological pain management, including paracetamol, nonsteroidal anti-inflammatory drugs (NSAIDs), acetylsalicylic acid, glyceryl trinitrate, opioids, ketamine, and pain-relieving gases. The most common routes of administration are intravenous and oral, although drugs such as fentanyl and sufentanil are available for intranasal use in some regions. Glyceryl trinitrate has the primary effect as vasodilator, though it can have an analgesic effect, so for the purpose of this study and in the context of chest pain, it is here being classed as an analgesic [16].

All 21 regional EMS organisations are connected to The Swedish Ambulance Registry, AmbuReg, which annually collects data from patient records. This includes mission response times, vital signs (i.e. respiratory rate, blood pressure, level of consciousness, and oxygen saturation), examinations, interventions, and treatments. At the time of this study, most Swedish EMS organisations (19/21) used the Rapid Emergency Triage and Treatment System (RETTS®) for patient assessment and triage. The RETTS is a validated tool that prioritises patients’ conditions based on vital signs and reasons for contact, assigning them to one of four priority levels: Red, Orange, Yellow, or Green. Red and Orange indicate the need for immediate medical attention, while Yellow and Green indicate that evaluation can be delayed [17, 18].

### Data collection

A dataset was retrieved from the AmbuReg, including all primary EMS missions with patient encounters from 1 January 2021 to 31 December 2022, and excluding inter-facility transfers. All data was anonymised when extracted from the registry and delivered to the research group without any possibility to identify specific individuals. The dataset was then filtered to include only cases meeting the study’s inclusion criteria: patients aged ≥ 18 years, alert (i.e., no altered level of consciousness), and RETTS ESS codes indicating pain-related conditions (5, chest pain; 6, abdominal pain; 13, joint pain without trauma; 14, back pain without trauma; 19, headache; 30, trauma/injury head, neck, throat, jaw or teeth; 31, trauma/injury, stomach, thorax, back or pelvis; 33, trauma/injury shoulder, arm or hand; 34, trauma/injury knee, hip, leg or foot; 38, multi-trauma and trauma alert activation; 42, physical abuse). I.e. ESS code including any of the words pain, ache, trauma, injury or abuse.

**Fig. 1:**
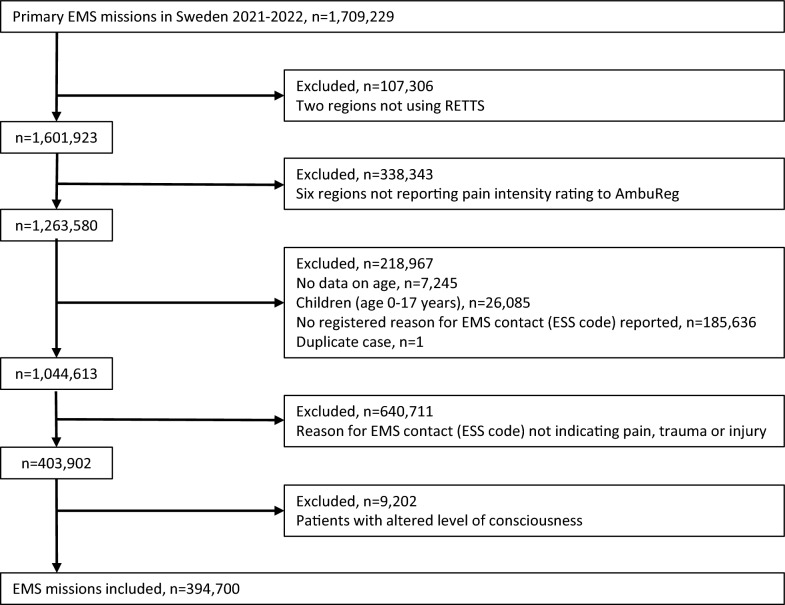
Study flow chart. *Legend*: EMS: Emergency Medical Services, RETTS: Rapid Emergency Triage and Treatment System, ESS: Emergency Signs and Symptoms

A flowchart summarising the inclusion and exclusion process is presented in Fig. 1. The main reasons for exclusion were that two regions did not use the RETTS, and six regions did not report pain intensity ratings to AmbuReg. When analysing the data from all 1,709,299 EMS missions (regardless of reason for EMS contact) registered in AmbuReg for 2021 and 2022, we found small differences in patient characteristics between the eight excluded regions and the thirteen regions included in the study. The median age in the included regions was 72 years old, compared to 69 years old in the excluded regions. In the included regions, 49.1% of the patients were male, compared to 49.9% in the excluded regions. The median time with patient (time at the scene plus transport time) was 42 min in the included regions, compared to 38 min in the excluded regions. There were varying proportions of missing data in the compiled cohort, including sex, RETTS priority, time on scene, patient transport time, time of day, and vital signs. Given the challenges of documentation in the EMS setting, such as stressful situations and limited personnel resources, some missing data were expected. The missing data are likely random in nature and are not believed to have affected the validity of the study [19]. However, missing data on time on scene and patient transport time, which were related to non-conveyance and therefore not missing at random, were excluded from the multivariate logistic regression analyses.

### Data analysis

For descriptive analyses, categorical variables are presented as frequencies and percentages, while continuous variables are reported as means with standard deviations and/or medians with interquartile ranges (Q1–Q3). Chi-square tests were used for dichotomous variables, and Kruskal–Wallis tests were applied to ordinal variables. Logistic regression was used for multivariate analyses and calculation of odds ratios (OR)/adjusted odds ratio (AOR). OR and AOR were used to quantify and describe the strength of the analysed associations and should not be interpreted as estimates of causal risk. A *p* value of < 0.001 was considered statistically significant. Statistical analyses were performed using IBM SPSS Statistics for Windows, version 29 (IBM Corp., Armonk, New York, USA).

Definitions of variables.

- *Median age (70 years)* was used as cut-off when analysing differences between younger and older patients.
- *NRS scores of 5–10* was considered as intense pain as NRS ≥ 5 is the highest known cut-off for initiating analgetic treatment in Swedish regional EMS guidelines
- *Pain intensity reduction* was defined as a reduction on the NRS between first and last NRS rating. If NRS score was only registered once this was assessed as no pain intensity reduction.
- *Drug types* were categorised based on The Anatomical Therapeutic Chemical codes (ATC codes) (Additional file 1).
- *Time on scene* was defined as time between EMS scene arrival and initiating patient transport.
- *Transport time* was defined as time between initiating patient transport and hospital arrival.

## Results

In total, 394,700 EMS missions were included, corresponding to 38% of all EMS missions for adult patients (n = 1,044,613) in the regions included in this study. Of these, 53.8% were women. The median age of the patients was 70 years old, with women being older than men (median age 73 vs. 67 years old). The highest triage priority level, RETTS Red, was assigned to 5.3% of cases. Median Time on scene and Patient transport time were 21 and 20 minutes, respectively (Table 1).

**Table 1:** Cohort overview

	All % (n)	Men % (n)	Women % (n)	Age < 70 years^a^ % (n)	Age ≥ 70 years^b^ % (n)
*Patient sex (1455 missing)*					
Men	46.2 (181,501)	100.0 (181,501)	–	53.3 (93,867)	46.7 (84,734)
Women	53.8 (211,744)	–	100.0 (211,744)	44.2 (93,604)	55.8 (118,140)
Age					
Mean (SD)	65 (22)	63 (21)	67 (21)	–	–
Median (Q25-Q75)	70 (50–82)	67 (48–80)	73 (51–84)	–	–
*EMS RETTS priority (129 missing)*					
RETTS red	5.3 (20,984)	6.7 (12,097)	4.2 (8793)	5.3 (10,164)	5.3 (10,820)
RETTS orange	37.0 (146,109)	38.2 (69,314)	36.0 (76,269)	31.7 (60,623)	42.1 (85,486)
RETTS yellow	42.0 (165,794)	40.4 (73,285)	43.4 (91,959)	45.7 (87,406)	38.6 (78,388)
RETTS green	15.5 (61,155)	14.6 (26,506)	16.2 (34,369)	17.2 (32,846)	13.9 (28,309)
RETTS blue	0.1 (529)	0.1 (236)	0.1 (290)	0.2 (297)	0.1 (232)
*Time with patient*					
Time on scene, minutes, median (Q25–Q75), (95,711 missing)	21 (15–29)	20 (14–28)	22 (16–30)	19 (13–26)	19 (13–26)
Patient transport time, minutes, median (Q25–Q75), (103,495 missing)	20 (10–33)	20 (11–33)	19 (10–32)	19 (10–32)	19 (10–32)
*Time of day (998 missing)*					
Daytime, 07:00–22:00	73.4 (288,830)	72.3 (130,886)	25.7 (54,312)	28.9 (55,127)	24.5 (49,745)
Nighttime, 22:01–06:59	26.6 (104,872)	27.7 (50,151)	74.3 (156,901)	71.1 (135,780)	75.5 (153,050)
*Vital signs*					
Breathing rate, breaths/minute, median (Q25–Q75), (15,593 missing)	18 (16–20)	18 (16–20)	18 (16–20)	18 (16–20)	18 (16–20)
Heart rate, beats/minute, median (Q25–Q75), (9,133 missing)	82 (72–95)	81 (70–94)	83 (74–95)	85 (74–98)	80 (70–92)
Systolic blood pressure, mmHg, median (Q25–Q75), (14,743 missing)	140 (125–160)	140 (126–158)	140 (125–160)	137 (122–151)	147 (130–165)

^a^ < median age, ^b^ ≥ median age

EMS: Emergency Medical Services, RETTS: Rapid Emergency Triage and Treatment System

### Pain assessment

Pain intensity was recorded using the Numeric Rating Scale (NRS) in 22.5% of cases, with a higher proportion of men than women having their pain assessed (23.6% vs. 21.6%, *p* < 0.001). Younger patients were more likely to have their pain intensity recorded compared to older patients (23.5% vs. 21.6%, *p* < 0.001). Of the patients whose pain was assessed, 57.4% reported high pain intensity (NRS 5–10). Women reported high pain intensity more often than men (59.7% vs. 54.9%, *p* < 0.001), and younger patients reported high-level pain more frequently than older patients (64.5% vs. 50.1%, *p* < 0.001) (Table 2). Most the common NRS rating was 0 (Additional file 2).

**Table 2:** Pain assessment

	All % (n)	Men^a^ % (n)	Women^a^ % (n)	*p* value^b^	Age < 70 years^c^% (n)	Age ≥ 70 years^d^ % (n)	*p* value^b^
All	100.0 (393,245)	100.0 (181,501)	100.0 (211,744)		100.0 (191,421)	100.0 (203,279)	
Recorded pain intensity	22.5 (88,899)	23.6 (42,857)	21.6 (45,842)	< 0.001	23.5 (45,031)	21.6 (43,868)	< 0.001
Pain intensity reduction during EMS mission^e^	26.2 (23,304)	26.1 (11,196)	26.3 (12,051)	0.579	28.8 (12,988)	23.5 (10,316)	< 0.001
NRS 0–4	42.6 (37,891)	45.1 (19,313)	40.3 (18,496)	< 0.001	35.5 (16,001)	49.9 (21,890)	< 0.001
NRS 5–10	57.4 (51,008)	54.9 (23,544)	59.7 (27,346)		64.5 (29,030)	50.1 (21,978)	

^a^Missing data on patient sex in 200 cases, ^b^Chi2, ^c^ < median age, ^d^ ≥ median age, ^e^among patients with pain intensity recorded at least once

### Pain reduction during EMS care

Among patients whose pain intensity was recorded, 26.2% reported a reduction in pain intensity during the EMS mission, i.e. a reduction on the NRS between first and last NRS rating. Median NRS reduction was 3 (Q25-Q75, 3–5). There was no significant difference between men and women in terms of pain reduction (26.1% vs. 26.3%, *p* = 0.579). However, younger patients reported a pain reduction more often than older patients (28.8% vs. 23.5%, *p* < 0.001) (Table 2).

### Administration of analgesics

Analgesics were administrated in 27.5% of the cases. Analgesics were more often provided if the patient had their pain intensity recorded (50.6% vs 20.8%) (*p* < 0.001), was young (28.5% vs 26.6%) (*p* < 0.001) or rated their pain as high-level (69.9% vs 24.7%) (*p* < 0.001). There was no difference between men and women regarding whether analgesics were administered or not. The most frequently used analgesics were opioids (15.6%), followed by paracetamol (8.8%) and glyceryl trinitrate (5.2%). Few patients (0.1%) required antidotes for adverse drug effects (Table 3). Among those receiving analgesics, 67.1% were treated with a single drug, 26.7% received two drugs, and 6.2% were administered more than two drugs.

**Table 3:** Medical treatment

	All % (n)	Recorded pain intensity % (n)			Patient sex^a^ % (n)			Age^b^ % (n)			Pain intensity^c^ % (n)		
	100.0 (394,700)	Recorded 22.5 (88,899)	Not recorded 77.5 (305,801)	*p* value^d^	Male 46.2 (181,501)	Female 53.8 (211,744)	*p* value^d^	Age < 70 48.5 (191,421)	Age ≥ 70 51.5 (203,279)	*p* value^d^	NRS ≤ 4 42.6 (37,891)	NRS > 4 57.4 (51,008)	*p* value^d^
*Analgesics*													
Analgesics administered (any kind)	27.5 (108,611)	50.6 (44,988)	20.8 (63,623)	< 0.001	27.5 (49,863)	27.6 (58,353)	0.549	28.5 (54,591)	26.6 (54,020)	< 0.001	24.7 (9,357)	69.9 (35,631)	< 0.001
Acetylsalicylic acid	1.9 (7604)	4.9 (4372)	1.1 (3232)	< 0.001	2.5 (4508)	1.5 (3079)	< 0.001	1.8 (3536)	2.0 (4068)	< 0.001	4.3 (1623)	5.4 (2749)	< 0.001
Acetaminophen/Paracetamol	8.8 (34,630)	15.2 (13,557)	6.9 (21,073)	< 0.001	8.1 (14,792)	9.3 (19,700)	< 0.001	9.0 (17,216)	8.6 (17,414)	< 0.001	8.2 (3111)	20.5 (10,446)	< 0.001
High-potency synthetic opioids^e^	1.5 (5947)	3.4 (2995)	1.0 (2952)	< 0.001	1.4 (2558)	1.6 (3375)	< 0.001	1.8 (3451)	1.2 (2496)	< 0.001	0.7 (278)	5.3 (2717)	< 0.001
Esketamine/Ketamine	1.6 (6282)	2.4 (22,124)	1.4 (4158)	< 0.001	1.2 (2261)	1.9 (3.991)	< 0.001	1.3 (2575)	1.8 (3707)	< 0.001	0.9 (324)	3.5 (1800)	< 0.001
Glyceryl trinitrate	5.2 (20,435)	10.8 (9559)	3.6 (10,876)	< 0.001	6.1 (10,997)	4.4 (9381)	< 0.001	4.6 (8866)	5.7 (11,569)	< 0.001	8.1 (3087)	12.7 (6472)	< 0.001
Nitrous oxide	0.1 (238)	0.1 (82)	0.1 (156)	< 0.001	0.1 (100)	0.1 (138)	0.200	0.1 (180)	0.0 (58)	< 0.001	0.0 (16)	0.1 (66)	< 0.001
Non-steroidal anti-inflammatory drug (NSAID)	2.1 (8109)	3.4 (3025)	1.7 (5084)	< 0.001	2.2 (4058)	1.9 (3994)	< 0.001	3.3 (6305)	0.9 (1804)	< 0.001	1.2 (451)	5.0 (2574)	< 0.001
Opioids^f^	15.6 (61,747)	31.1 (27,656)	11.1 (34,091)	< 0.001	15.6 (28,335)	15.7 (33,204)	0.549	0.7 (1403)	0.5 (1062)	< 0.001	8.7 (3304)	47.7 (24,352)	< 0.001
Other/unspecified analgesics	0.6 (2465)	0.8 (737)	0.6 (1728)	< 0.001	0.6 (1012)	0.7 (1444)	< 0.001	16.1 (30,789)	15.2 (30,958)	< 0.001	0.3 (117)	1.2 (620)	< 0.001
Antidote administered (any kind)	0.1 (206)	0.1 (104)	0.0 (102)	< 0.001	0.1 (97)	0.1 (106)	0.642	0.1 (99)	0.1 (107)	0.900	0.0 (14)	0.2 (90)	< 0.001
Sedatives/Muscle relaxants/Anaesthetics	1.5 (6075)	2.9 (2549)	1.2 (3526)	< 0.001	1.5 (2643)	1.6 (3415)	< 0.001	1.7 (3335)	1.3 (2740)	< 0.001	0.9 (334)	4.3 (2215)	< 0.001

^a^1455 missing data on patient sex, ^b^categorised by median age, ^c^305,801 no recorded pain intensity, ^d^chi2 ^e^alfentanil/fentanyl/sufentanil, ^f^oxycodone/morphine/ketobemidone

In a multivariate regression analysis, pain intensity recording was the factor most strongly associated with the administration of analgesics (AOR 3.896, 99.9% CI 3.794–4.001). Female sex was also positively associated with analgesic administration (AOR 1.039, 99.9% CI 1.014–1.065). EMS mission during daytime (AOR 0.959, 99.9% CI 0.933–0.986) was negatively associated with analgesic administration (Table 4).

**Table 4:** Factors associated with analgesic treatment

	Treatment with analgesics	
	Adjusted odds ratio	Confidence interval, 99.9%
Female sex	1.039	1.014–1.065
Older age	1.000	0.999–1.000
Recorded pain intensity	3.896	3.794–4.001
Daytime	0.959	0.933–0.986

Multivariate logistic regression based on 392,250 complete cases

### Causes and types of pain

Pain related to illness was more common than pain caused by trauma/injury (60.4% vs. 39.6%). Older patients were more likely to report pain caused by lower extremity trauma, while younger patients were more frequently affected by multi-trauma or physical abuse. Chest pain and abdominal pain were the most common reasons for contacting the EMS (28.6% and 21.9%, respectively).

Patients with pain related to illness more often rated their pain as high-level compared to those with trauma-related pain (60.9% vs. 49.8%). Pain intensity was most frequently recorded for patients with abdominal pain (27.5%) and chest pain (26.4%), while it was least recorded for patients with headache (17.6%) and physical abuse (7.8%) (Additional file 3).

### Factors associated with pain intensity

Factors associated with higher pain intensity (NRS > 4) included female sex (AOR 1.30, 99.9% CI 1.24–1.36), higher breathing rate (AOR 1.12, 99.9% CI 1.12–1.13), and higher systolic blood pressure (AOR 1.003, 99.9% CI 1.002–1.004). Increasing age was associated with lower odds of reporting high pain intensity (AOR 0.981, 99.9% CI 0.980–0.982). Heart rate showed no significant association with pain intensity (Table 5).

**Table 5:** Factors associated with NRS > 4

	Adjusted odds ratio	Confidence interval (99.9%)
Older age	0.981	0.980–0.982
Female sex	1.301	1.241–1.364
Breathing rate^a^	1.124	1.116–1.132
Heart rate^a^	0.999	0.998–1.000
Systolic blood pressure^a^	1.003	1.002–1.004

Multivariate logistic regression based on 86,477 complete cases

^a^Continuous variable based on first registered value by the EMS

## Discussion

This observational study, based on data from a nationwide ambulance registry, found that 38% of the EMS population had a pain-related condition caused by illness or injury, with only 27.5% receiving treatment with analgesics. Most patients were elderly women, who reported higher pain intensity compared to men. Breathing rate and systolic blood pressure showed a weak but significant association with higher pain intensity.

An important finding was that fewer than a quarter of patients had their pain assessed using a validated pain assessment scale, which may have resulted in undertreatment of pain. Patients whose pain was assessed were significantly more likely to receive analgesics, a finding supported by previous studies indicating that pain assessment increases the likelihood of pain relief [20]. Furthermore, men were more likely than women to have their pain assessed, a pattern previously observed in Sweden, particularly among men presenting with chest pain. Pain assessment also occurred more frequently in younger patients than in older patients, possibly reflecting differences in healthcare priorities or communication challenges across age groups. This discrepancy in pain assessment has been highlighted in earlier studies as well [21].

Interestingly, younger patients showed a reduction in pain intensity, in terms of a lowered NRS rating during the EMS mission, more often than older patients (28.8% vs. 23.5%). Suggesting that pain management may be more effective in younger populations. However, the low overall adherence to pain assessment protocols in the EMS indicates a need for improvement. Similar findings in other studies emphasise the critical role of guideline adherence in achieving effective pain management [22]. Effective prehospital pain management hinges on patient empowerment, meeting expectations, and a holistic approach combining pharmacological and non-pharmacological methods. Seamless transitions and collaboration between prehospital and hospital care, supported by shared guidelines, education, and feedback systems, are essential for quality care [23].

The majority of patients with registered pain intensity in this study reported a high pain intensity (NRS 5–10), with women rating their pain as high-level more often than men (59.7% vs. 54.9%). This can be partially explained by different reasons for contacting the EMS, a finding that is also supported by other studies [24]. Another explanation is that women may experience and express pain differently [25]. Women generally report higher pain sensitivity and a greater prevalence of chronic pain conditions than men, and this also applies to acute settings. These differences are influenced by both biological and psychosocial factors [26]. These gender differences in pain perception may increase the risk of women developing persistent pain following acute episodes [27]. Despite these differences in reported pain intensity, no significant gender disparities were observed in this study regarding the effectiveness of pain management and pain reduction.

Pain intensity above NRS 4 was a strong predictor for analgesic administration, reflecting adherence to national guidelines [28]. This suggests that pain intensity was a determining factor in whether the patient received an analgesic [22]. However, the exclusive reliance on NRS scores as a trigger for analgesic use warrants reconsideration. While NRS scores provide a useful baseline, pain management decisions should be more nuanced, considering patient-specific factors such as pain tolerance, previous experience with analgesics, and the risk of adverse effects. Balancing effective pain relief with the risk of overtreatment, particularly with opioids, remains critical [29].

Only 27.5% of patients received analgesics, a lower rate compared to recent Swedish studies reporting 40.7% pain treatment among trauma patients [30]. This discrepancy may be due to differences in study design or the inclusion of non-trauma cases in the current study. The most frequently used analgesics were opioids (15.6%) followed by paracetamol (8.8%) and glyceryl trinitrate (5.2%). Among those receiving analgesics 26.7% received two drugs, and 6.2% were administered more than two drugs. Although the Swedish EMS has access to a broad range of analgesics, the findings highlight the need for improvement in pain management practices. Combining pharmacological options to enhance pain relief has been proposed in earlier research [31], but caution is required in older patients with multimorbidity or polypharmacy to avoid adverse drug reactions [32].

Less than one percent of patients received an antidote, suggesting that the frequency of adverse events can be interpreted as low. Similar frequencies of adverse events have previously been interpreted as safe regarding the administration of analgesics in the EMS [33].

Multivariate analysis revealed that pain assessment had the greatest impact on the likelihood of analgesic administration (AOR 3.53), followed by patients’ sex. Women were more likely to receive analgesics, consistent with their higher reported pain levels, as observed in other studies [34].

Patients without trauma-related pain were more likely to have their pain documented and to report high pain intensity, especially for abdominal and chest pain. Patients with lower extremity injuries were overrepresented among older adults and women, probably due to hip fractures. This may indicate limitations in the NRS as a pain assessment tool. In a study comparing the prevalence and intensity of pain among groups with chest pain, abdominal pain, and pain following hip injury, NRS and free-text descriptions were mostly used by patients experiencing chest and abdominal pain [9]. For patients with pain following hip injury, the most frequently used tool was a behaviour-related scale (BRS), consistent with another study showing that pain was assessed with BRS in two out of three patients with hip pain [7]. These findings suggest that pain assessment instruments need further development.

The modest but significant association observed in this study between vital signs and pain intensity is supported by findings from other studies. However, the limited strength of the correlation and its low clinical precision suggest that this finding may not hold significant clinical relevance [8].

## Strengths and limitations

A key strength of this study is its large sample size, derived from a nationwide registry that includes data from nearly all Swedish EMS organisations. To the authors’ knowledge, this is the largest study on EMS pain assessment and management to date. However, the study has several limitations. Its retrospective observational design limits the ability to draw causal conclusions. Furthermore, data from eight regions were excluded due to not using the RETTS or not reporting pain intensity ratings to AmbuReg, which may introduce selection bias. As differences between included and excluded regions were minor, the findings may be considered applicable to the Swedish EMS in general, although local variation should be acknowledged. Generalisation to an international context should be done with caution, given differences in EMS staffing and available medical treatments.

Furthermore, there are potential confounders that could affect the results. For example, pain intensity was more commonly recorded for non-trauma/injury patients compared to those with trauma. This could reflect a true difference, but may also be the result of confounding factors, such as older patients being more frequently subjected to trauma, and older age being associated with a lower likelihood of pain intensity being recorded.

The biased distribution of missing data may also affect the multivariate logistic regression analysis of factors associated with increased pain. The analysis was based on 86,477 complete cases out of 394,700, with missing pain intensity registration being the most common cause for exclusion. Therefore, the results of the analysis should be interpreted with caution, as the missing data may be biased, potentially influencing the findings.

## Conclusions

This two-year cohort study highlights significant deficiencies in recorded pain assessment and management in the Swedish EMS. Only 22.5% of the patients had their pain assessed with a validated scale, while 27.5% received analgesics, although pain-related conditions were a common reason for contacting the EMS. The findings indicate a lack of systematic pain assessment which puts many patients at risk of insufficient pain relief. To improve the quality of care, it is crucial to implement more consistent and systematic methods for pain assessment and management. Future efforts should focus on the education and training of EMS personnel, as well as the development of guidelines, pain assessment instruments and protocols to ensure that all patients receive the pain relief they need and are entitled to.

## Supplementary Information

Additional file 1

Additional file 2

Additional file 3

## Data Availability

The dataset generated and analysed during the current study is not publicly available. Data are however available from the corresponding author upon reasonable request and if approved by AmbuReg.

## Abbreviations

AOR: Adjusted odds ratio
AmbuReg: The Swedish Ambulance Registry
ATC: Anatomical Therapeutic Chemical
BRS: Behaviour Rating Scale
EMT: Emergency Medical Technician
EMS: Emergency Medical Services
ESS: Emergency Signs and Symptoms
NSAID: Non-Steroidal Anti-Inflammatory Drugs
NRS: Numeric Rating Scale
OR: Odds ratio
RETTS: Rapid Emergency Triage and Treatment System
RN: Registered nurse
VAS: Visual analogue scale

## References

[1] Ibsen S, Lindskou TA, Nickel CH, Kløjgård T, Christensen EF, Søvsø MB. Which symptoms pose the highest risk in patients calling for an ambulance? A population-based cohort study from Denmark. Scand J Trauma Resusc Emerg Med. 2021;29(1):59. 33879211 10.1186/s13049-021-00874-6 PMC8056716

[2] Friesgaard KD, Riddervold IS, Kirkegaard H, Christensen EF, Nikolajsen L. Acute pain in the prehospital setting: a register-based study of 41.241 patients. Scand J Trauma Resusc Emerg Med. 2018;26(1):53. 29970130 10.1186/s13049-018-0521-2 PMC6029421

[3] Ariès P, Montelescaut E, Pessey F, Danguy des Déserts M, Giacardi C. Pre-hospital emergency medicine: pain control. Lancet. 2016;387(10020):747. 26906662 10.1016/S0140-6736(16)00325-1

[4] Ferri P, Gambaretto C, Alberti S, Parogni P, Rovesti S, Di Lorenzo R, et al. Pain management in a prehospital emergency setting: a retrospective observational study. J Pain Res. 2022;15:3433–45. 36324866 10.2147/JPR.S376586 PMC9621014

[5] Eichinger M, Robb HDP, Scurr C, Tucker H, Heschl S, Peck G. Challenges in the PREHOSPITAL emergency management of geriatric trauma patients—a scoping review. Scand J Trauma Resusc Emerg Med. 2021;29(1):100. 34301281 10.1186/s13049-021-00922-1 PMC8305876

[6] Ismail AK, Abdul Ghafar MA, Shamsuddin NS, Roslan NA, Kaharuddin H, Nik Muhamad NA. The assessment of acute pain in pre-hospital care using verbal numerical rating and visual analogue scales. J Emerg Med. 2015;49(3):287–93. 26022936 10.1016/j.jemermed.2015.02.043

[7] Wennberg P, Möller M, Sarenmalm EK, Herlitz J. Evaluation of the intensity and management of pain before arrival in hospital among patients with suspected hip fractures. Int Emerg Nurs. 2020;49: 100825. 32029418 10.1016/j.ienj.2019.100825

[8] Andersson JO, Nasic S, Herlitz J, Hjertonsson E, Axelsson C. The intensity of pain in the prehospital setting is most strongly reflected in the respiratory rate among physiological parameters. Am J Emerg Med. 2019;37(12):2125–31. 30718118 10.1016/j.ajem.2019.01.032

[9] Magnusson C, Carlström M, Lidman N, Herlitz J, Wennberg P, Axelsson C. Evaluation and treatment of pain in the pre-hospital setting. A comparison between patients with a hip injury, chest pain and abdominal pain. Int Emerg Nurs. 2021;56:100999. 33765527 10.1016/j.ienj.2021.100999

[10] Galinski M, Ruscev M, Gonzalez G, Kavas J, Ameur L, Biens D, et al. Prevalence and management of acute pain in prehospital emergency medicine. Prehosp Emerg Care. 2010;14(3):334–9. 20507221 10.3109/10903121003760218

[11] Bounes V, Barniol C, Minville V, Houze-Cerfon CH, Ducassé JL. Predictors of pain relief and adverse events in patients receiving opioids in a prehospital setting. Am J Emerg Med. 2011;29(5):512–7. 20825821 10.1016/j.ajem.2009.12.005

[12] Bendall JC, Simpson PM, Middleton PM. Prehospital vital signs can predict pain severity: analysis using ordinal logistic regression. Eur J Emerg Med. 2011;18(6):334–9. 21407079 10.1097/MEJ.0b013e328344fdf2

[13] Jennings PA, Cameron P, Bernard S. Measuring acute pain in the prehospital setting. Emerg Med J. 2009;26(8):552–5. 19625547 10.1136/emj.2008.062539

[14] Larsson G, Axelsson C, Hagiwara MA, Herlitz J, Klementsson H, Troëng T, et al. Epidemiology of patients assessed for trauma by Swedish ambulance services: a retrospective registry study. BMC Emerg Med. 2024;24(1):11. 38191306 10.1186/s12873-023-00924-5 PMC10775538

[15] Lindström V, Bohm K, Kurland L. Prehospital care in Sweden From a transport organization to advanced healthcare. Notfall Rettungsmedizin. 2015;18:107–9.

[16] Kim KH, Kerndt CC, Adnan G, et al. Nitroglycerin. [Updated 2023 Jul 31]. In: StatPearls [Internet]. Treasure Island (FL): StatPearls Publishing; 2025. https://www.ncbi.nlm.nih.gov/books/NBK482382/. 29494004

[17] Magnusson C, Herlitz J, Axelsson C. Patient characteristics, triage utilisation, level of care, and outcomes in an unselected adult patient population seen by the emergency medical services: a prospective observational study. BMC Emerg Med. 2020;20(1):7. 32000684 10.1186/s12873-020-0302-x PMC6993445

[18] Wireklint SC, Elmqvist C, Parenti N, Göransson KE. A descriptive study of registered nurses’ application of the triage scale RETTS©; a Swedish reliability study. Int Emerg Nurs. 2018;38:21–8. 29326039 10.1016/j.ienj.2017.12.003

[19] Kaji AH, Schriger D, Green S. Looking through the retrospectoscope: reducing bias in emergency medicine chart review studies. Ann Emerg Med. 2014;64(3):292–8. 24746846 10.1016/j.annemergmed.2014.03.025

[20] Rahman NH, Ananthanosamy C. The display effects of patients’ self-assessment on traumatic acute pain on the proportion and timing of analgesics administration in the emergency department. Int J Emerg Med. 2014;7:36. 25635196 10.1186/s12245-014-0036-1 PMC4306068

[21] Davies B. Healthcare priorities: the “Young” and the “Old.” Camb Q Healthc Ethics. 2023;32(2):174–85. 36352770 10.1017/S0963180122000299 PMC10425921

[22] Ruhe MM, Veldhuis LI, Azijli-Abdelloui K, Schepers T, Ridderikhof ML. Prehospital analgesia in suspected hip fracture patients: adherence to national prehospital pain management guidelines. Eur J Trauma Emerg Surg. 2024;50(3):937–43. 37957364 10.1007/s00068-023-02385-8

[23] Whitley GA, Wijegoonewardene N, Nelson D, Curtis F, Ortega M, Siriwardena AN. Patient, family member, and ambulance staff experiences of prehospital acute pain management in adults: a systematic review and meta-synthesis. J Am Coll Emerg Phys Open. 2023;4(2): e12940. 10.1002/emp2.12940 PMC10086522 37056718

[24] Wibring K, Lingman M, Herlitz J, Pettersson H, Lerjebo A, Bång A. Clinical presentation in EMS patients with acute chest pain in relation to sex, age and medical history: prospective cohort study. BMJ Open. 2022;12(8): e054622. 35940838 10.1136/bmjopen-2021-054622 PMC9364405

[25] Fillingim RB, King CD, Ribeiro-Dasilva MC, Rahim-Williams B, Riley JL. Sex, gender, and pain: a review of recent clinical and experimental findings. J Pain. 2009;10(5):447–85. 19411059 10.1016/j.jpain.2008.12.001 PMC2677686

[26] Paller CJ, Campbell CM, Edwards RR, Dobs AS. Sex-based differences in pain perception and treatment. Pain Med. 2009;10(2):289–99. 19207233 10.1111/j.1526-4637.2008.00558.x PMC2745644

[27] Burns JW, Janssen I, Lillis T, Mulcahy M, Purim-Shem-Tov YA, Bruehl S, et al. The transition from acute to persistent pain: the identification of distinct trajectories among women presenting to an emergency department. Pain. 2020;161(11):2511–9. 32569094 10.1097/j.pain.0000000000001960 PMC10853846

[28] SLAS. Behandlingsriktlinjer - Smärtbehandling allmänt: Sveriges Ledningsansvariga Ambulansläkare i Samverkan; 2021 Available from: https://slas.infosynk.se/category/behandling/Do79XB2UP4SnzYwOS8mk.

[29] van Dijk JF, Kappen TH, Schuurmans MJ, van Wijck AJ. The relation between patients’ NRS pain scores and their desire for additional opioids after surgery. Pain Pract. 2015;15(7):604–9. 24735082 10.1111/papr.12217

[30] Larsson G, Axelsson C, Hagiwara MA, Herlitz J, Magnusson C. Characteristics of a trauma population in an ambulance organisation in Sweden: results from an observational study. Scand J Trauma Resusc Emerg Med. 2023;31(1):33. 37365663 10.1186/s13049-023-01090-0 PMC10294300

[31] Sobieraj DM, Baker WL, Martinez BK, Miao B, Hernandez AV, Coleman CI, et al. Comparative effectiveness of analgesics to reduce acute pain in the prehospital setting. 2019. 31509367

[32] Al-Qurain AA, Gebremichael LG, Khan MS, Williams DB, Mackenzie L, Phillips C, et al. Prevalence and factors associated with analgesic prescribing in poly-medicated elderly patients. Drugs Aging. 2020;37(4):291–300. 32016823 10.1007/s40266-019-00742-0

[33] Wennberg P, Pakpour A, Broström A, Karlsson K, Magnusson C. Alfentanil for pain relief in a Swedish emergency medical service—an eleven-year follow-up on safety and effect. Prehosp Emerg Care. 2024. 10.1080/10903127.2024.2363509. 38830199 10.1080/10903127.2024.2363509

[34] Safdar B, Heins A, Homel P, Miner J, Neighbor M, DeSandre P, et al. Impact of physician and patient gender on pain management in the emergency department–a multicenter study. Pain Med. 2009;10(2):364–72. 18992042 10.1111/j.1526-4637.2008.00524.x

