# Supplementary material for: Pain assessment and management of adult patients in the Swedish EMS: a nationwide registry study
Source: Scand J Trauma Resusc Emerg Med. 2025 Feb 5;33:22. doi: 10.1186/s13049-025-01333-2 (PMC11796019; doi:10.1186/s13049-025-01333-2)
Supplement: Supplementary file 1 — Additional file 1 [file 13049_2025_1333_MOESM1_ESM.docx]

**Additional file 1. Matrix of ATC-codes and drug categorisation**

| ATC code | Drug category |
| --- | --- |
| B01AC06 | Acetylsalicylic acid |
| C01D | Glyceryl trinitrate |
| C01DA | Glyceryl trinitrate |
| C01DA02 | Glyceryl trinitrate |
| M01A | Non-steroidal anti-inflammatory drug |
| M01AB | Non-steroidal anti-inflammatory drug |
| M01AB05 | Non-steroidal anti-inflammatory drug |
| M01AB15 | Non-steroidal anti-inflammatory drug |
| M01AE01 | Non-steroidal anti-inflammatory drug |
| M01AE02 | Non-steroidal anti-inflammatory drug |
| M01AH05 | Non-steroidal anti-inflammatory drug |
| M03BB03 | Sedatives/ Muscle relaxants/ Anaesthetics |
| N0 | Sedatives/ Muscle relaxants/ Anaesthetics |
| N01 | Sedatives/ Muscle relaxants/ Anaesthetics |
| N01A | Sedatives/ Muscle relaxants/ Anaesthetics |
| N01AH | Sedatives/ Muscle relaxants/ Anaesthetics |
| N01AH01 | High-potency synthetic opioids |
| N01AH02 | High-potency synthetic opioids |
| N01AH03 | High-potency synthetic opioids |
| N01AX03 | Esketamine, Ketamine |
| N01AX1 | Sedatives/ Muscle relaxants/ Anaesthetics |
| N01AX10 | Other analgesics |
| N01AX14 | Esketamine, Ketamine |
| N01AX63 | Nitrous oxide |
| N02 | Other analgesics |
| N02A | Opioids |
| N02AA | Opioids |
| N02AA01 | Opioids |
| N02AA05 | Opioids |
| N02AA59 | Opioids |
| N02AB01 | Opioids |
| N02B | Other analgesics |
| N02BA01 | Acetylsalicylic acid |
| N02BE | Acetaminophen/Paracetamol |
| N02BE0 | Acetaminophen/Paracetamol |
| N02BE01 | Acetaminophen/Paracetamol |
| N02BG09 | Other analgesics |
| N05B | Sedatives/ Muscle relaxants/ Anaesthetics |
| N05BA01 | Sedatives/ Muscle relaxants/ Anaesthetics |
| N05BA04 | Sedatives/ Muscle relaxants/ Anaesthetics |
| N05BB01 | Sedatives/ Muscle relaxants/ Anaesthetics |
| N05C | Sedatives/ Muscle relaxants/ Anaesthetics |
| N05CD | Sedatives/ Muscle relaxants/ Anaesthetics |
| N05CD09 | Sedatives/ Muscle relaxants/ Anaesthetics |
| N05CF01 | Sedatives/ Muscle relaxants/ Anaesthetics |
| S01HA03 | Other analgesics |
| V03 | Antidote |
| V03A | Antidote |
| V03AB06 | Antidote |
| V03AB1 | Antidote |
| V03AB15 | Antidote |
| V03AB25 | Antidote |
